# Microwave-Assisted Lignin Wet Peroxide Oxidation to C_4_ Dicarboxylic Acids

**DOI:** 10.1021/acs.iecr.1c05004

**Published:** 2022-03-04

**Authors:** Carlos A. Vega-Aguilar, Carina Costa, Maria Filomena Barreiro, Alírio E. Rodrigues

**Affiliations:** † Laboratory of Separation and Reaction Engineering—Laboratory of Catalysis and Materials (LSRE-LCM), Department of Chemical Engineering, Faculdade de Engenharia, Universidade do Porto, Rua Dr. Roberto Frias s/n, 4200-465 Porto, Portugal; ‡ Centro de Investigação de Montanha—CIMO, Instituto Politécnico de Bragança, Campus de Santa Apolónia, 5300-253 Bragança, Portugal

## Abstract

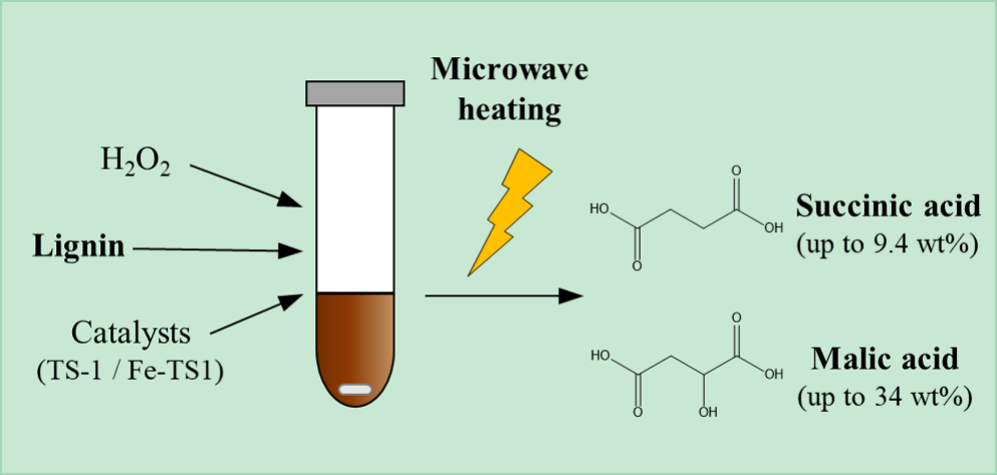

Innovative methodologies, such as microwave-assisted reaction, can help to valorize lignin with higher productivity and better energy efficiency. In this work, microwave heating was tested in the wet peroxide oxidation of three lignins (Indulin AT, Lignol, and *Eucalyptus globulus* lignins) as a novel methodology to obtain C_4_ dicarboxylic acids. The effect of temperature, time, and catalyst type (TS-1 or Fe-TS1) was evaluated in the production of these acids. The TS-1 catalyst improved succinic acid yield, achieving up to 9.4 wt % for Lignol lignin. Moreover, the microwave heating specifically enhanced Lignol conversion to malic acid (34 wt %), even without catalyst, showing to be an attractive path for the future valorization of organosolv lignins. Overall, compared to conventional heating, microwave heating originated a rapid lignin conversion. Nevertheless, for prolonged times, conventional heating led to better results for some target products, e.g., malic and succinic acids.

## 1 Introduction

Lignin is formed by three main monomeric units, namely, *p*-hydroxyphenyl (H), guaiacyl (G), and syringyl (S), linked by aryl ether and C–C bonds, forming a three-dimensional complex matrix.^1,2^ Pulping processes (e.g., kraft, sulfite, soda, and organosolv) are used to extract lignin, generating an annual amount of 130 million tons of kraft lignin.^3−5^ The final lignin has a chemical structure and properties depending not only on the plant origin but also on the pulping process.

As an underutilized renewable feedstock, lignin can be valorized to added-value compounds through various chemical and thermochemical approaches.^6,7^ Oxidative depolymerization produces aromatic and aliphatic compounds by cleaving the main bonds present in lignin.^8,9^ It usually follows a radical pathway leading to three main reactions: side-chain cleavage, aromatic ring cleavage, and condensation reactions.^10,11^ The production of aromatic aldehydes (e.g., vanillin and syringaldehyde) has been widely studied due to their direct valorization.^12,13^ Recently, an increased interest in C_4_ dicarboxylic acids (C_4_-DCA) like succinic, maleic, and fumaric acids, arose given their current industrial use and prospects as future biomass-based building blocks.^14,15^ It is necessary to use harsh reaction conditions to achieve an aromatic ring-opening reaction, namely, strong oxidants (usually H_2_O_2_, O_3_, or organic peroxides) assisted by heterogeneous catalysis.^5^

Traditionally, oxidative conversion of lignin has been carried out using conventional heating (CH), usually by applying high temperatures and pressures, and toxic or expensive solvents and catalysts. Nevertheless, there has been an increasing interest in nonconventional methods, providing milder conditions.^16^ Microwave (MW) is a nonionizing radiation that does not interact with the chemical bonds, producing heat by enhancing the kinetic energy of the molecules, depending on three properties: electric, dielectric, and magnetic.^17^ Heat can propagate through two mechanisms, ionic conduction and bipolar rotation. The first one refers to the movement of ions trying to follow the electric current, causing shocks with other molecules and heat releasing. The second mechanism, with a strong relationship with the dielectric properties of the compounds, is caused by the fast alignment of polar molecules with the electric field, causing friction and heat release.^18^

MW heating has several advantages compared with conventional heating, such as faster heat transfer, shorter reaction times, nonlocalized heating avoiding surface overheating, low vessel heat loss, ability to promptly turn on/off the system, capability to pressurize the system for temperatures above the boiling point, and more efficient use of energy. However, some disadvantages are the cost and equipment availability, nonuniform heating of nonhomogeneous materials, low penetration of MW radiation, and changes of the dielectric properties with temperature, affecting material’s affinity to MW.^17,18^

MW heating has been used in lignin conversion processes, especially in pyrolysis reactions, generating bio-oils rich in phenolic compounds,^17,19,20^ which generally require an additional upgrade to high added-value compounds.^21^ Lignin oxidation using MW has been introduced as a new way to improve the production of added-value compounds, such as aromatic aldehydes and acids (e.g., vanillin and syringaldehyde). Lignin model compounds oxidation has been studied to understand how MW influences the reaction pathways. In several works, the MW oxidation of lignin model compounds was faster than with conventional heating (CH).^22,23^ An electrodeless lamp combined with MW and H_2_O_2_ caused guaiacol photooxidation to, mainly, formic, acetic, and oxalic acids, with yields dependent on pH, time, and concentration of guaiacol and H_2_O_2_.^24^ Zhu et al.^25^ studied C_α_–C_β_ bond cleavage of two lignin model dimers, finding that MW improved its efficiency, especially for phenolic dimers and organosolv lignins, releasing more aromatic monomers. The oxidation of lignin models with β-O-4 bonds using H_2_O_2_ and CuO, both with MW and CH systems, showed the formation of the same reaction products, but at different rates, since some oxidation steps were accelerated by MW and others not.^26^ For example, the oxidation of vanillin to vanillic acid was accelerated, but the demethylation step and the ring-opening reaction were not. To the best of our knowledge, this work was the first study reporting C_4_-DCA production, including succinic, maleic, malic, and fumaric acids, from lignin model oxidation using MW.

Oxidation of real lignin samples showed different results, depending on the used lignin and experimental conditions. MW peroxide oxidation of soda lignin leads to a better degradation of the high-molecular-weight fractions to lower fragments compared to CH.^27^ Nevertheless, MW also facilitated the re-condensation reactions. The used temperature, time, and oxidant load affected the degradation and re-condensation. In a subsequent study, the authors verified that acidic conditions, high temperatures, and a correct amount of H_2_O_2_ were needed to maximize degradation.^28^ They also observed that MW oxidation gave rise to fewer products, mainly aliphatic alkanes, alcohols, acids, and esters, due to the cleavage of aromatic rings and deprivation of the side chains. In contrast, a more complex mixture of products, composed mainly of aromatic compounds, was observed with CH. The MW oxidation of sulfonated lignin using KOH produced phenolic compounds that differed from the ones obtained by CH-assisted oxidation.^29^

Heterogeneous catalysts can be used with MW heating since they are good MW absorbers, heating very quickly, and reaching, in some cases, a temperature higher than that of the liquid phase due to hot spots.^30^ Alkaline-fractionated lignin depolymerization using different catalysts (CuO, Cu(OH)_2_, Fe_2_O_3_, Cu_2_O) in MW-assisted peroxide oxidation yielded vanillin and acetosyringone as the main products, with carboxylic acids being formed at low quantities.^31^ It was confirmed that Cu^2+^ promoted the cleavage of lignin side chains and ether linkages, while Fe^3+^ enhanced H_2_O_2_ oxidation performance and the yield in monophenols.^32^ Moreover, peroxide oxidation of three lignins using CuSO_4_ showed that the conversion was affected by the oxidant concentration, catalyst type, reaction time, and high temperatures. CuSO_4_ has an essential role in ^•^OH radical production, and the MW accelerated this reaction, producing aromatic compounds (acids and aldehydes) as the main products.^33^

In this study, three lignins [Indulin AT (IAT), Lignol (EOL), and *Eucalyptus globulus* lignin (EKL)] were oxidized using titanium silicalite-1 (TS-1) and Fe-TS1-modified catalyst, and H_2_O_2_ as the oxidant. TS-1 improved the hydrogen peroxide conversion in MW-mediated *n*-hexane oxyfunctionalization,^34^ while enhancing the C_4_-DCA yields in lignin peroxide oxidation, giving rise up to 11.3% succinic acid and 19.5 wt % malic acid,^35^ which are similar or even better yields compared to other reported catalysts, such as chalcopyrite^36,37^ and Fe^3+^ in O_2_,^38^ with the advantage of being an industrial and widely available catalyst.^39^ Given the importance of MW as a more efficient way to heat the reaction medium, the combined study of TS-1 catalysis with MW is proposed in this work. Moreover, the MW heating results were compared with CH experiments to evaluate the advantages/disadvantages of the heating source in the C_4_-DCA production from lignin.

## 2 Materials and Methods

### 2.1 Materials

All chemical reagents were purchased from commercial sources and used without further purification: hydrogen peroxide solution (Fluka, >30% p.a.), sulfuric acid (Chem-labs, 95–97% p.a.), sodium hydroxide (Merck, p.a.), FeSO_4_·7H_2_O (Panreac, 97% p.a.), deuterated dimethyl sulfoxide (DMSO-*d*_6_; VWR, 99.80%), *N*,*N*-dimethylformamide (VWR, ≥99.9%) and lithium chloride (VWR, AnalaR NORMAPUR). Catalyst TS-1 (ref #: MSTS1001, lot number: 130117; H^+^ cation) was acquired from ACS Materials, LLC. Fe-TS1 was obtained by modifying the original TS-1 by wet impregnation, as reported in Vega-Aguilar et al.^40^

The three lignins were Indulin AT (IAT), commercialized by MeadWestvaco Corporation; a lignin isolated in May 2021 from an industrial black liquor obtained from a Portuguese *E. globulus* Kraft pulping mill (The Navigator Company, Portugal) (EKL); and a lignin produced by an ethanol organosolv process from *E. globulus* (EOL), supplied by Lignol Innovations, Canada.

### 2.2 Lignin Characterization

Lignin characterization includes the determination of the major components (ashes, carbohydrates, and acid-soluble and acid-insoluble lignin), gel permeation chromatography (GPC), and Fourier transform infrared spectroscopy in attenuated total reflectance mode (ATR-FTIR). All the described characterizations were carried out for EKL, a lignin isolated exclusively for this work. IAT and EOL characterization had been reported in previous works.^35,41^ Quantitative ^13^C NMR characterization was carried out for IAT and EKL lignins. Quantitative ^13^C NMR analysis for EOL lignin was previously reported by Costa et al.^41^

Ashes were quantified by incinerating 0.5 g of lignin at 600 °C until a constant mass was achieved. Carbohydrates content was determined by performing an acid methanolysis of the lignin. After cooling, pyridine and sorbitol (internal standard) were added, and the solution was evaporated under reduced pressure. The methanolysates were derivatized using trimethylchlorosilane. Then, quantification was performed using gas chromatography-flame ionization detector (GC-FID). A complete description of the procedure can be found elsewhere.^42^ Insoluble lignin was quantified by dissolving the lignin sample in an alkaline solution until complete dissolution was achieved, then acidified with H_2_SO_4_ 2 mol/L until pH 2; heating at 40 °C to coagulate the lignin, then followed by centrifugation at 3500 rpm for 30 min. The insoluble lignin was dried at 100 °C overnight and weighted. The acid-soluble lignin content was considered as 100% – ashes content (%) – carbohydrates content (%) – acid-insoluble lignin content (%).

Quantitative ^13^C NMR analysis of lignins was performed using a Bruker AVANCE III 400 spectrometer, operating at 400 MHz, at 45 °C for 72 h. Lignin samples (170 mg) were dissolved in 0.5 mL of deuterated dimethyl sulfoxide (DMSO-*d*_6_). The quantitative conditions used for ^13^C NMR measurements were: simple one-dimensional (1D) pulse sequence, recycling time of 12 s, 1400 scans, and 1D sequence with power gated coupling using 90° flip angle. More details about the applied method can be found elsewhere.^43^

GPC was used to evaluated lignin molecular weight and polydispersity index. A Shimadzu Ultra-Fast Liquid Chromatography (UFLC) equipment, equipped with a Diode Array Detector (280 nm) was used with two Agilent columns in series: an OligoPore column (300 mm × 7.5 mm, 6 μm nominal particle size) followed by a MesoPore column (300 mm × 7.5 mm, 3 μm nominal particle size). Before this arrangement, an OligoPore precolumn (300 mm × 7.5 mm) was used. Analysis was performed at 70 °C, using dimethylformamide with 0.5 wt % LiCl, at 0.8 mL/min. Calibration was done using polystyrene (PS) standards in the molecular weight range between 162 and 50 000 g/mol (calibration curve can be consulted in Figure S1). More details about the applied method can be found elsewhere.^43^

ATR-FTIR measurements were carried out using a JASCO FT/IR-6800 spectrometer (JASCO Analytical Instruments), equipped with a MIRacle Single Reflection (ZnSe crystal plate; PIKE Technologies). The analysis was performed by co-adding 256 scans in the range 4000–700 cm^–1^, using a resolution of 4 cm^–1^. IAT and EOL measurements were previously reported by Vega-Aguilar et al.^35^

### 2.3 Oxidation Procedure

#### 2.3.1 Microwave-Assisted Oxidation

Lignins were oxidized using a Biotage Initiator+ microwave reactor. A lignin solution (2.5 mL, 10 g/L, pH 7.0, dissolved in water) was placed inside a 2–5 mL microwave vial with a stirring bar, then added with 0.250 mL of a 30 wt % H_2_O_2_ solution and 2.5 mg of catalyst when applied. The reactors were pre-stirred at 780 rpm for 30 s, the magnetron turned on, and the heating started using the Very high absorption level, reaching the desired temperature after ca. 90 s. Stirring was maintained during the oxidation to avoid hot spots in the solution that would produce localized overoxidation. At the end of the reaction time, the microwave vial was immediately cooled with compressed air, reaching 40 °C around 2 min after the end of the reaction. The effect of temperature (140–170 °C), reaction time (0–3 h), catalyst type (TS-1, Fe/TS-1), and lignin type (Indulin AT, Lignol, and *E. globulus* kraft lignins) was studied. The fixed values for H_2_O_2_ load, catalyst load, lignin concentration, and pH were selected based on previous works for lignin peroxide oxidation using conventional heating in a batch reactor, published elsewhere.^35^ Experiments were done in duplicate, and results were expressed as average with the respective error bars.

Oxidation using only Fe^2+^ as a homogeneous catalyst (Fenton’s reagent) was applied to IAT lignin to evaluate if a combined effect of the Fe atoms and the TS-1 structure justifies the differences between TS-1 and Fe-TS1, or if it is just caused by the Fe atoms placed at the catalyst surface.

#### 2.3.2 Conventional Heating Oxidation

Conventional heating oxidation experiments for IAT and EOL, used for comparison purposes in Section 3.3, were previously reported in Vega-Aguilar et al.,^35^ where a complete description of the experimental methods and quantitative analysis can be found. The conventional heating system consisted of a Teflon vessel placed inside a preheated steel reactor, placed over a heating plate, and protected with insulation material. Additional experiments were performed for IAT lignin using the same methodology, namely, for 30 min reaction time at 140 °C in the presence of the TS-1 catalyst.

### 2.4 Quantification of Oxidized Lignin

Oxidized lignin samples were acidified to pH ∼ 2, heated at 40 °C to coagulate the acid-insoluble lignin, then centrifuged at 3500 rpm for 30 min. The acidic supernatant was used for carboxylic acid and acid-soluble lignin quantification. The insoluble lignin was resolubilized in an alkaline solution. Acid-soluble and insoluble lignin solutions were analyzed by UV spectrophotometry at 240 nm and quantified based on a calibration curve with the acid-soluble and insoluble lignin obtained from the original lignin, respectively. Lignin conversion was calculated as the sum of the acid-insoluble and the acid-soluble lignins. Calibration curves for acid-soluble and insoluble lignins can be found in Figure S2.

### 2.5 Quantification of Carboxylic Acids

Carboxylic acids were quantified by high-performance liquid chromatography (HPLC) analysis using a Shimadzu UFLC, equipped with a Diode Array Detector (210 nm), a refractive index detector (RI), and a Phenomenex Rezex ROA H+ column (300 mm × 7.8 mm) and precolumn (50 mm × 7.8 mm). The analysis was performed at 50 °C using isocratic mode (4 mmol/L H_2_SO_4_) at a 0.5 mL/min flow rate and an injection volume of 20 μL. Carboxylic acids were identified by comparison with retention times and quantified using calibration curves of individual standards. Before injection, samples were acidified, diluted as needed, and filtered through a 0.22 μm pore-size filter. Calibration curves can be found in Table S1. Quantified acids are expressed as C_4_-DCA (sum of succinic, malic, maleic, fumaric, and tartaric acids).

## 3 Results and Discussion

### 3.1 Lignin Characterization

To better understand the composition and chemical structure of the studied lignins, they were characterized by FTIR, ^13^C NMR, GPC, and by quantifying the major components (acid-soluble and insoluble lignin, carbohydrate, and ash contents). This information is helpful to better understand the relationship between lignin structure and peroxide oxidation behavior.

As seen in Table 1, lignins IAT and EOL are commercial products with high purity (i.e., high lignin content and low content of contaminants (carbohydrates and ashes)). On the contrary, EKL, a lignin isolated from a black liquor obtained from a Portuguese pulping plant using *E. globulus* as feedstock, presented high content of carbohydrates and ashes (i.e., it is a low-purity lignin). This sample was isolated at the lab scale, resulting in difficulties in the washing step of the precipitated lignin. Even after three washing cycles with ultrapure water, plenty of the ashes remained in the lignin, mainly Na_2_SO_4_. However, the EKL was included in this work to evaluate the oxidative conversion of low-purity lignins obtained from pulp and paper production.

**Table 1:** Composition of Lignins (IAT, EOL, and EKL), Presented in % w/w_lignin_

lignin	acid-insoluble lignin content (% w/w)	acid-soluble lignin content (% w/w)^a^	carbohydrate content (% w/w)	ash content (% w/w)
IAT	92.1	1.93	2.43	3.54
EOL	95.3	3.2	1.41^(●)^	0.11^(●)^
EKL	65.0	3.7	3.2	28.1

a Marked data (^●^) were obtained from ref (41). Acid-soluble lignin is obtained by difference.

Gel permeation chromatography was used to compare the molecular weight of EKL with the previously reported lignins IAT and EOL,^35^ as seen in Table 2. EOL presented the higher *M*_w_ and *M*_n_, as well as the higher polydispersity (*Đ*_M_). IAT and EKL showed lower *M*_w_ and *M*_n_. As both IAT and EKL are kraft lignins, it is expected that the harsh extraction process conditions might reduce the lignin size compared to the milder extraction conditions of the organosolv process (EOL). As the IAT is a softwood lignin (high percentage of G units), the available sites at the C_5_ position allow an easy condensation during the extraction process, which corroborates the high degree of condensation comparatively with the other two lignins (hardwood lignins). This fact is also associated with the β-O-4 content, given that this bond is mainly cleaved in the extraction process. Naturally, this bond has an abundance of 45–50% (softwood) or 60–62% (hardwood).^21^ Due to the extraction process conditions, this value decreases, but EOL still keeps a high content,^41^ while kraft lignins show lower values, especially IAT.

**Table 2:** Structural Properties of the Studied Lignins (IAT, EOL, and EKL)^a^

lignin	*M*_w_ (g/mol)	*M*_n_ (g/mol)	*Đ*_M_	S/G/H ratio	DC (%)	β-O-4 units (per 100 Ar)
IAT	12 285^(▲)^	4288^(▲)^	2.86^(▲)^	18:77:05	58	20
EOL	14 458^(▲)^	4496^(▲)^	3.22^(▲)^	70:30:0^(●)^	35^(●)^	34^(●)^
EKL	11 595	4243	2.73	69:28:03	26	29

a Marked data (^●^) and (^▲^) were obtained from refs (41) and (35), respectively.

**Figure 1:**
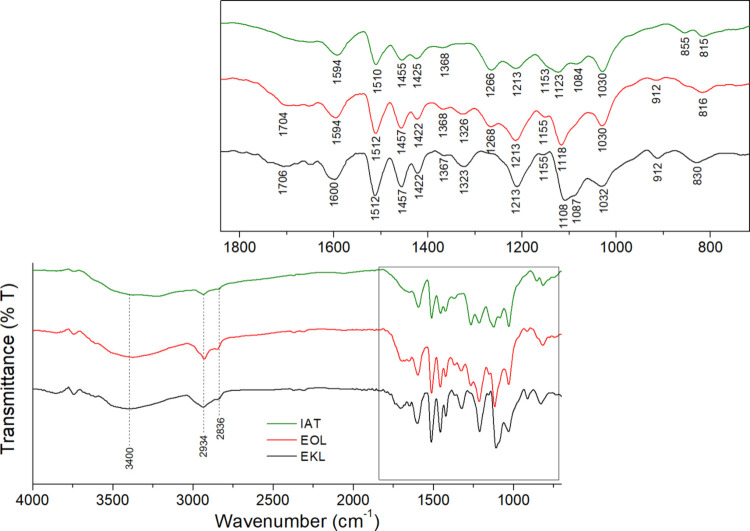
ATR-FTIR analysis for the studied lignins (IAT and EOL spectra reprinted with permission from ref (35). Copyright 2021, Elsevier).

The ATR-FTIR analysis (Figure 1) evidences the characteristic peaks of lignin as reported by Faix^44^ and Cateto et al.^45^ EKL showed the same bands reported previously for IAT and EOL,^35^ namely, the ones associated with OH stretching (3400 cm^–1^), C–H stretching of methyl and methylene groups (2934 and 2836 cm^–1^), and vibrations associated with the aromatic rings (1594, 1512, and 1422 cm^–1^). IAT presented only softwood characteristic vibrations (for guaiacyl units) at 1266 and 855 cm^–1^, while EOL and EKL also showed signals for syringyl groups in the 1323–1326 and 816–830 cm^–1^ regions. Also, the 1030 cm^–1^ band was more intense in softwood lignins, while bands at 1456 and 1108–1123 cm^–1^ were more intense in hardwood lignins. All these bands are associated with C–H deformations. Finally, the band at 1213 cm^–1^, associated with a combined effect of C–C, C–O, and C=O stretching, was more intense in hardwood lignins.

A ^13^C NMR analysis was performed to get information concerning lignin structure, including chemical bonds, for IAT and EKL and compared to previously reported EOL data.^41^ The assignments were based on Costa et al.’s^41^ work, and the results are included in Table 3. EOL proved to be the lignin with higher content of β-O-4 bonds,^41^ comparatively with EKL and IAT. However, all lignins showed lower β-O-4 values than the expected ones, probably due to a more vigorous depolymerization process, while the β-5 + β–β units were in the expected ranges.^21^ The condensation reactions can easily occur in softwood lignins (IAT), given the available C_5_ position in G units.

**Table 3:** Assignments and Quantification of Structures/Linkages and Functional Groups, Identified by ^13^C NMR (Number Per Aromatic Ring)^a^

	amount (number/Ar)		
lignin	IAT	EOL^(●)^	EKL
β-5 and β–β structures (δ 51.0–53.8 ppm)	0.07	0.10	0.13
aromatic OCH_3_ (δ 54.3–57.3 ppm)	0.82	1.40	1.36
C_γ_ in β-O-4 structures without C_α_=O (δ 59.3–60.8 ppm)	0.13	0.26	0.14
C_γ_ in β-5 and β-O-4 structures with C_α_=O; C_γ_ in β-1 (δ 62.5–63.8 ppm)	0.05	0.07	0.16
C_α_ in β-O-4 structures; C_γ_ in pinoresinol/syringaresinol and β–β structures (δ 70.0–76.0 ppm)	0.26	0.34	0.79
C_β_ in β-O-4 structures; C_α_ in β-5 and β–β structures (δ 80.0–90.0 ppm)	0.27	0.44	0.42
aromatic C_Ar_-H (δ 103.0–123.0 ppm)	2.19	1.95	2.02
aromatic C_Ar_-C (δ 123.0–137.0 ppm)	1.72	1.75	1.77
aromatic C_Ar_-O (δ 137.0–156.0 ppm)	2.05	2.30	2.19
C_4_ in H units (δ 157.0–162.0 ppm)	0.04	0.00	0.03
CHO in benzaldehyde structures (δ 191.0–192.0 ppm)	0.02	0.04	0.03
CHO in cinnamaldehyde structures (δ 193.5–194.5 ppm)	0.01	0.04	0.03
CO in aldehydes and ketones (δ 195.0–210.0 ppm)	0.20	0.47	0.41

a Marked data (^●^) were obtained from ref (41).

In contrast, hardwood lignins (EKL and EOL) have methoxyl groups in both C_3_ and C_5_ positions, diminishing the possibility of condensation reactions. This relation can also be confirmed in the aromatic OCH_3_ groups, which are higher for EOL and EKL and lower for IAT. Another important fact is that EOL shows no *p*-hydroxyphenyl units (H) while EKL shows 3% and IAT 5% of H units. The H units are more difficult to be oxidized to DCA due to the absence of methoxyl groups since these substituents activate the aromatic ring to further oxidation.^46^

### 3.2 Microwave-Assisted Oxidation

MW-assisted reactions can enhance lignin oxidation, but a relationship exists between the degradation process and the reaction conditions (pH, temperature, oxidant load, and time).^28^ The effect of temperature, time, and catalyst type in the lignin oxidation toward C_4_-DCA was evaluated in this work and discussed next.

#### 3.2.1 Effect of Temperature

Lignin oxidation depends significantly on the used lignin type, especially in what concerns the origin and pulping method.^35^ As a starting point, IAT was selected to evaluate the best temperature for MW-assisted oxidation since this lignin originated the highest succinic acid when CH was used.

**Figure 2:**
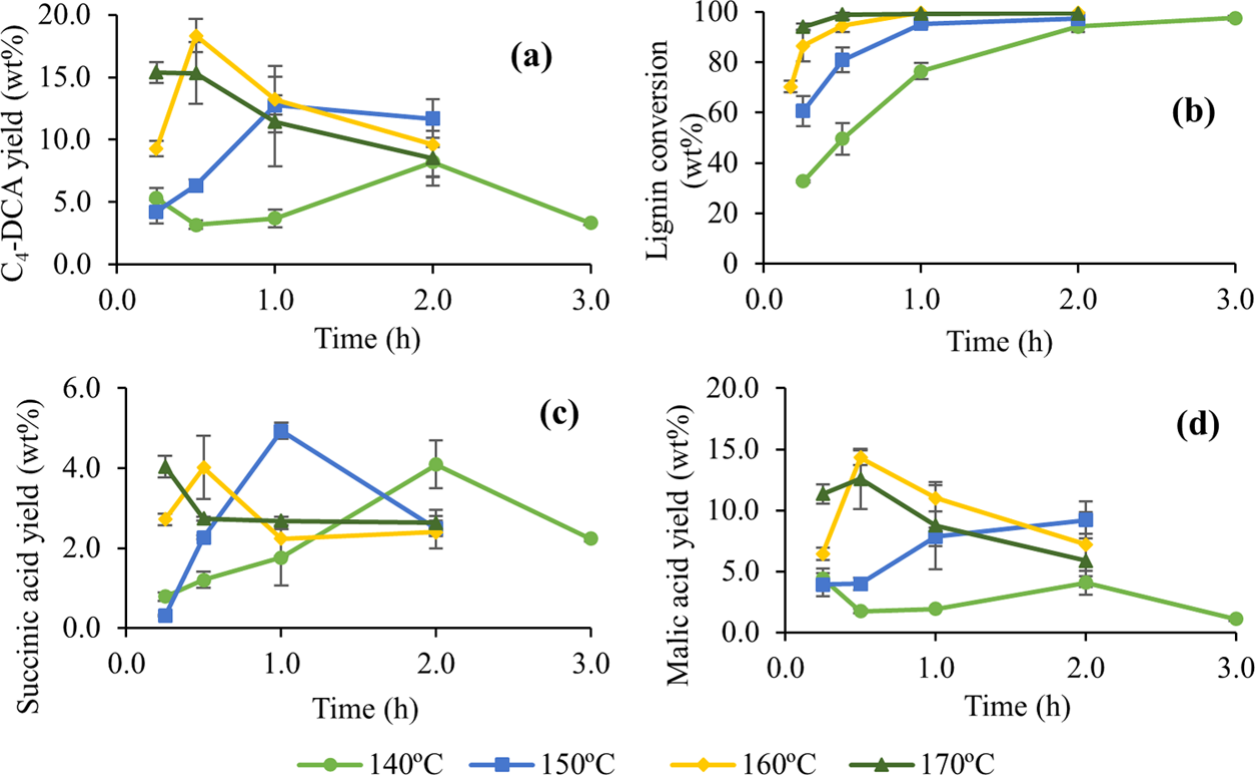
Noncatalyzed microwave-assisted oxidation of IAT: Effect of temperature on (a) C_4_-DCA yield and (b) lignin conversion, and yields for (c) succinic acid and (d) malic acid.

For IAT noncatalyzed oxidation, the temperature strongly affected the C_4_-DCA yields (sum of succinic, malic, maleic, fumaric, and tartaric acids). In Figure 2a, the maximum C_4_-DCA yield moved toward shorter times and higher values as the temperature increases. The reaction using the lowest temperature (140 °C) took 2.0 h to reach the highest yield (8.2 wt %), while at 160 °C, the C_4_-DCA yield was 18.3 wt % just after 30 min. The maximum yield for the highest tested temperature (170 °C) was achieved after 15 min, then decreased, indicating that the acids were degraded after this point. The same behavior was observed for the other tested temperatures, showing a slow decrease after the C_4_-DCA maximum yield. Succinic and malic acids shared the same increasing-decreasing maximum yield behavior, as shown in Figure 2c,d, respectively. These acids represented more than 98% of the quantified C_4_-DCA.

**Figure 3:**
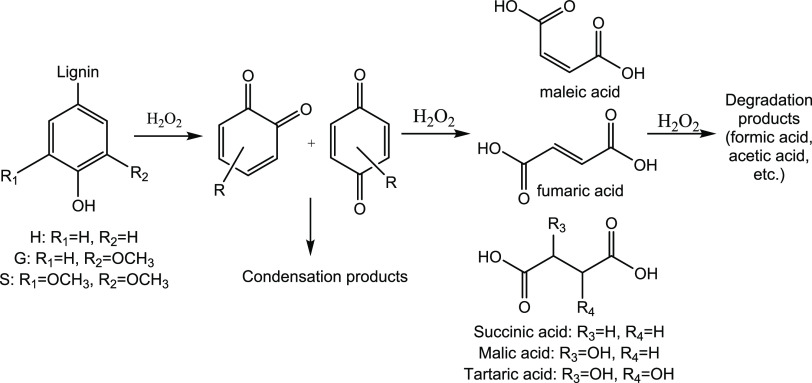
Reaction scheme for lignin peroxide oxidation through ring-opening reactions to produce C_4_-DCA (H: *p*-hydroxyphenyl units; G: guaiacyl units; S: syringyl units).

Interestingly, succinic acid yield did not significantly increment with temperature, with the maximum yield achieved at shorter times. On the contrary, the yield increased with the temperature for malic acid, going from 4.4% (140 °C) to 14.3% (160 °C). Probably, at 170 °C malic acid yield did not increase due to the fast degradation of the already-produced acids. Also, higher temperatures increased the degradation to low-molecular-weight compounds, like formic and acetic acids (Figure S3a,b, respectively). Succinic and malic acids were degraded to formic and acetic acids, as seen in Figure 3, confirming the decrease of the C_4_ acids after the maximum. Formic acid was rapidly degraded to CO_2_ at high temperatures, while acetic acid remained stable without decreasing its content even at high temperatures. As acetic acid was produced in low quantities as a byproduct, the catalytic effect of peracetic acid (formed by the mixture of acetic acid and H_2_O_2_) was not observed. Probably, succinic acid did not achieve a higher yield since it was converted to malic acid and other compounds under oxidative conditions,^47,48^ corroborated by the increase in malic acid yield.

As expected, lignin conversion strongly depended on temperature, as shown in Figure 2b. At least 2 h were needed to achieve a lignin conversion higher than 97% at 140 °C, while at 170 °C, it only took 30 min, confirming the important effect of temperature in lignin oxidation to added-value compounds. This effect has already been corroborated in other publications regarding lignin oxidation in MW reactors.^26,28,29^

The experiments performed at 170 °C showed some technical problems due to the rapid reaction inside the vessel, namely, the peroxide oxidation released O_2_ from H_2_O_2_ disproportion and CO_2_ from complete lignin mineralization, causing a rapid pressure increase exceeding the safety recommendations (max. 5 bar/s). For these reasons, the studied temperature in the following experiments was 160 °C. Also, when water is used as the solvent it responded to microwave radiation in a controlled way if the equipment is correctly tuned, allowing proper heating of the solution. Other organic polar solvents absorb more efficiently the microwave radiation, but they heat faster, enhancing the H_2_O_2_ disproportion and reducing the oxidative power of the reaction. Water is used as a green solvent in microwave given its characteristics such as ready availability at a low cost, nontoxicity, environmental benignity, and the possibility of working at high pressures and temperatures.^49^

#### 3.2.2 Catalytic Effect: TS-1 and Modified Fe-TS1

**Figure 4:**
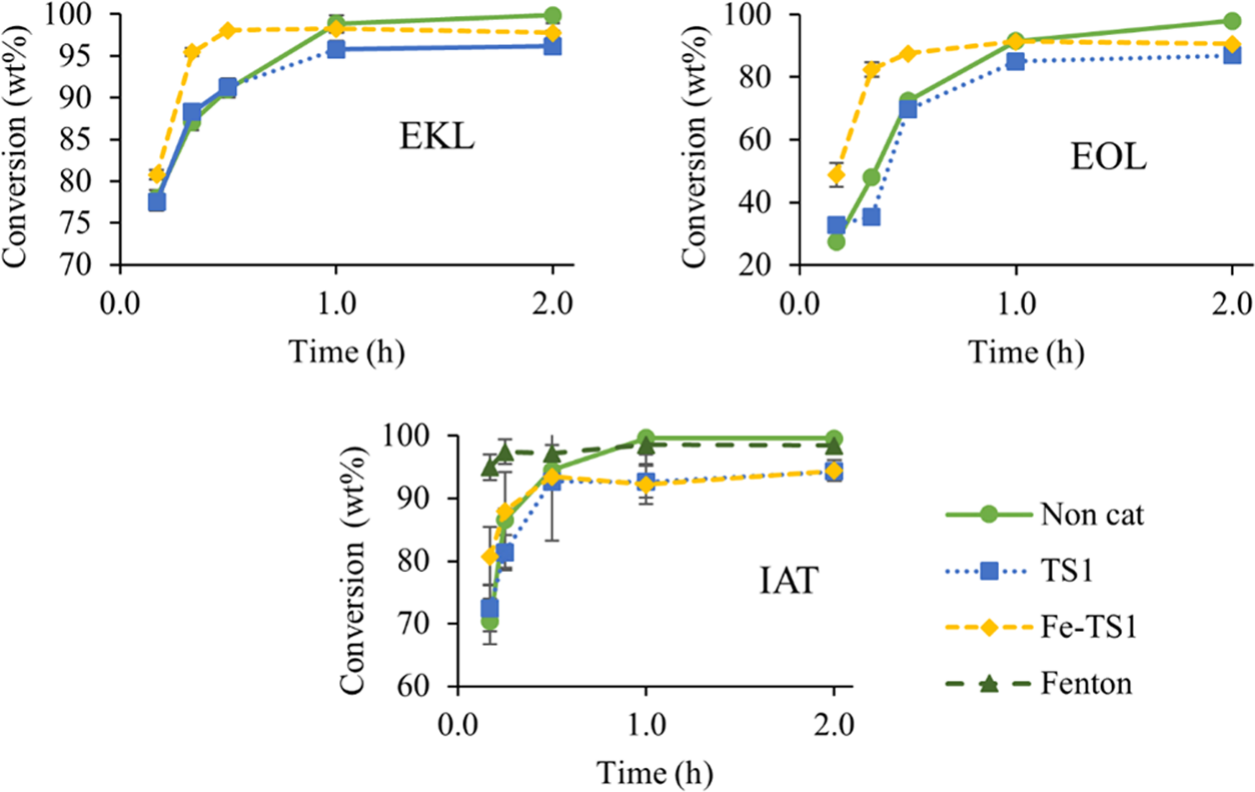
Evolution of lignin conversion through time in the microwave-assisted oxidation for EKL, EOL, and IAT lignins (*T* = 160 °C).

Catalysts have been used to improve the conversion and selectivity of lignin oxidations toward added-value compounds.^7^ Titanium silicalite-1 (TS-1) was used for lignin peroxide oxidation,^35^ and a higher succinic acid yield was obtained when the catalyst was present. To evaluate if the same effect is observed when using MW, the three lignins were oxidized at 160 °C under three catalytic conditions, namely, when using TS-1 catalyst, Fe-modified TS-1 catalyst, which were compared with the noncatalyzed reaction. Lignin conversion was slightly lower when TS-1 was used, as seen in Figure 4, while the noncatalyzed reaction reached complete conversion after 2.0 h for the three tested lignins. The same behavior was previously observed when conventional heating was applied.^35^ It has been reported that TS-1 stabilizes the hydrogen peroxide, avoiding very severe oxidation and slowing the ^•^OH radicals’ release.^34^ Interestingly, when Fe-TS1 was used in EKL and EOL, the conversion was higher at shorter times, indicating that the Fe atoms in the catalyst structure enhanced the hydroxyl radical formation, increasing lignin depolymerization to lower-molecular-weight compounds.^32^

Succinic and malic acids were the main C_4_ acids obtained after the oxidation, with very low yields for fumaric and maleic acids (<0.10 and <0.20 wt %, respectively), and no tartaric acid was detected. Even though maleic acid was not found in significant quantities, it is a fundamental intermediate after ring-opening reaction to produce malic and succinic acid.^46^ Due to its high reactivity in oxidative conditions associated with its labile double bond, it was found in very low amounts since it is converted quickly to malic acid given the conditions of high temperatures and high peroxide loads,^46^ compared to other works where milder conditions were used.^39^

**Figure 5:**
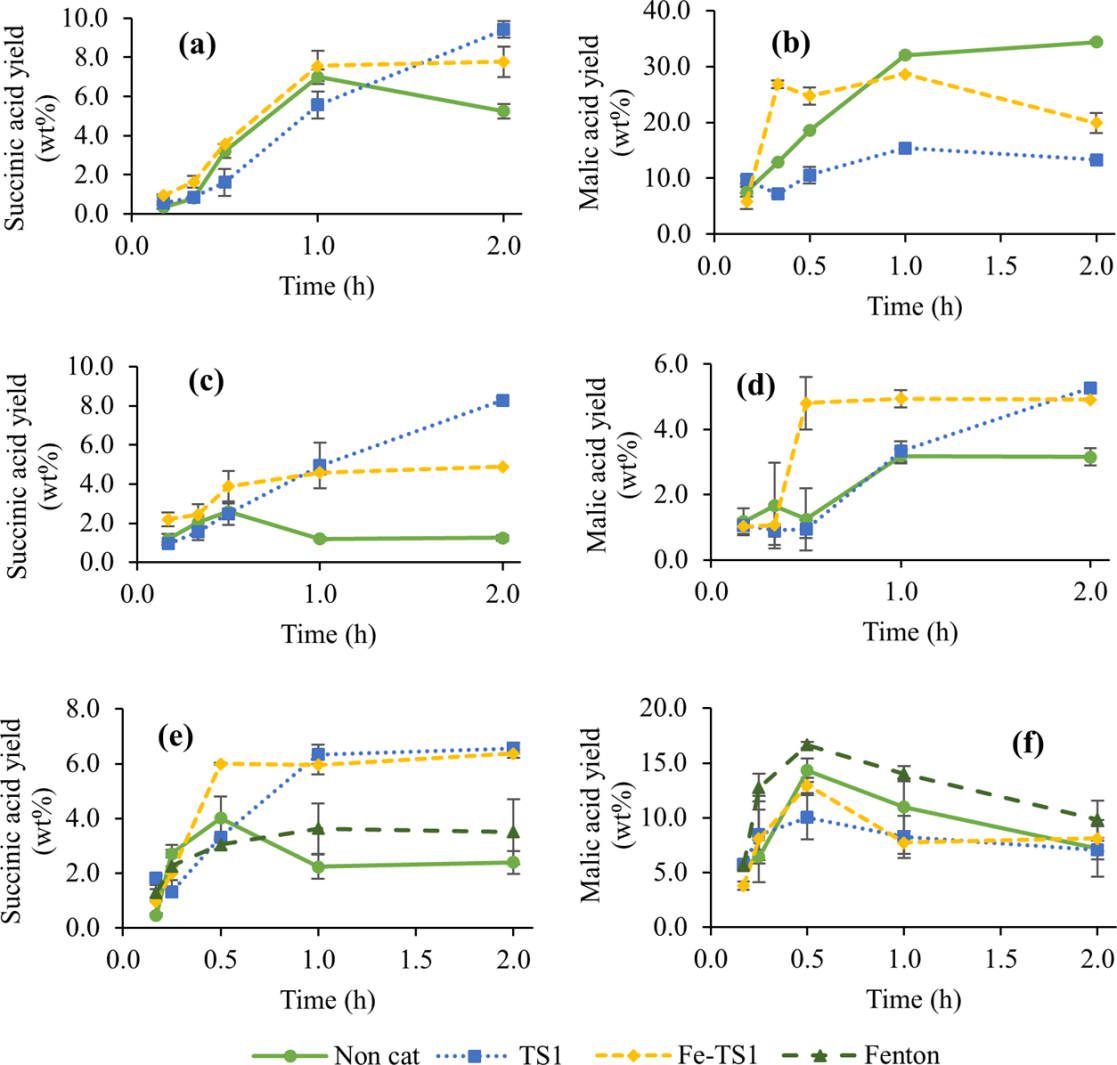
Evolution of C_4_-DCA yields through time in microwave-assisted oxidation. Succinic acid (left column) and malic acid (right column) yields for (a, b) EOL, (c, d) EKL, and (e, f) IAT (*T* = 160 °C).

As seen in Figure 5, the noncatalyzed reaction showed a maximum for succinic acid yield, then decreased slowly. The time to achieve the maximum yield depended on the used lignin. For the TS-1 catalyzed reaction, a continuously increasing yield was observed; only IAT reached a plateau after 1.0 h (yield −6.3%). The EOL lignin gave rise to the highest succinic acid yields (9.4% for TS-1, 7.8% for Fe-TS1, 5.2% for noncatalyst, as seen in Figure 5a). The kraft lignins (EKL and IAT) resulted in lower yields, strictly dependent on the used catalyst (Figure 5c,e, respectively). For EKL and IAT, the succinic acid yield was more than twice of the corresponding noncatalyzed reaction. With the Fe-TS1 catalyst, the succinic acid production was faster in the first 30 min for the studied lignins, but after 1.0 h, the nonmodified TS-1 led to similar yields or even higher. This information confirms that the modified Fe-TS1 only accelerated succinic acid production in the first minutes of the reaction, compared to TS-1, not increasing the maximum yield. Also, it can be seen that the catalytic oxidations avoid over-oxidations of the succinic acid, confirming milder reaction conditions. In comparison, the noncatalytic oxidation leads to harsher conditions degrading the succinic acid. Moreover, even though EKL was a lignin with lower purity, an interesting amount of succinic acid was produced, especially in the presence of the catalyst, showing that the impurities present in the lignin did not influence the conversion to succinic acid.

EOL lignin produced the highest yield for malic acid, reaching up to 34% for the noncatalyzed reaction (Figure 5b), while EKL was the lignin originating the lowest yield (max. 5.2%, Figure 5d). For EKL and EOL, the malic acid yield increased rapidly with Fe-TS1. For IAT, the maximum yield occurred after 0.5 h, then decreased; while for EKL, the Fe-TS1 maximum yield was also achieved at 0.5 h, but after 2.0 h the TS-1 catalyzed reaction produced a higher yield. Both noncatalyzed and Fe-TS1 catalyzed reactions behaved similarly in EOL, while the lower yields were observed when using TS-1.

EOL originated higher yields of succinic and malic acids, but also acetic and formic acids (Figure S4). Overall, quantified acids after 1.0 h reaction for EOL accounted up to 56.9% (noncatalyzed), 47.9% (TS-1), and 54.5% (Fe-TS1). EOL is an organosolv lignin with a mild pulping process and higher content of easily cleaved ether bonds (as seen in Table 2). Therefore, in EOL, the oxidant is mainly used to break the ether bonds, releasing the available hydroxyl groups in the phenolic ring, facilitating the aromatic ring opening to produce the C_4_-DCA. As oxidation is more efficient with this lignin, the amount of C_4_-DCA is higher. The easier degradation to C_4_-DCA was also observed when conventional heating was used.^35^ For EOL, a similar succinic acid yield was achieved in the catalyzed and noncatalyzed reactions, together with a higher malic acid yield in the noncatalyzed reaction, which shows that for an organosolv lignin, the MW facilitates the oxidation even without catalyst.

Fenton oxidation leads to a faster IAT lignin conversion (Figure 4), given that Fe ions enhance the oxidation ability of H_2_O_2_, breaking the lignin structure faster, opening the aromatic ring, and producing oxidized low-molecular-weight compounds.^32^ It was noted in Figure 5e that Fe-TS1 produced more succinic acid in 0.5 h, but the TS-1 reaction reached a similar yield after 1.0 h. The Fenton’s catalyzed reaction behaved similarly to the noncatalyzed reaction, resulting in low yields, while both TS-1/Fe-TS1 catalysts resulted in higher yields. Regarding malic acid, the best yield was achieved for the Fenton’s reaction, followed closely by the noncatalyzed reaction and the Fe-TS1. In all cases, the highest malic yield acid was achieved in the first 30 min, then decreasing. Once maleic acid is formed, it can be oxidized to malic acid or hydrated to succinic acid.^46^ As the TS-1/Fe-TS1 catalysts behaved similarly, resulting in high succinic acid yield, and Fenton’s reagent behaves similarly to the noncatalyzed reaction for malic acid yield, it can be inferred that the TS-1 and the Fe-TS1 catalysts preferably follow the pathway to succinic acid. On the contrary, Fenton’s catalyst and the noncatalyzed reaction favor the oxidation of maleic acid to malic acid. Thus, TS-1 and Fe-TS1 allow a more selective oxidation toward succinic acid production.

It has been reported that since H_2_O_2_ adsorbs in the tetrahedral Ti active sites of the TS-1 form, Ti-OOH species are formed, which have an increased nucleophilic attack capability compared to the H_2_O_2_ molecules.^50^ These species later interact with the organic compounds close to the catalyst surface. Since the radicals are released in a controlled way, the lignin is oxidized by opening the aromatic ring and releasing the dicarboxylic acids (mainly maleic acid). However, as the acids are released to the aqueous medium, it becomes more difficult to interact with the acid catalyst surface to hydrate the double bond to form succinic acid. In the noncatalyzed reaction, the free ^•^OH radicals are produced directly by the disproportion of the H_2_O_2_ in the aqueous medium, becoming available to attack the lignin structure, the already formed products, or combine with other radicals. The Fe atoms in the Fe-TS1 structure disproportioned H_2_O_2_ instantly to free radicals, having the Fe-TS1 catalyst a mixed behavior between the stability of the TS-1 oxidation and the quick oxidation of the noncatalyzed reaction, producing succinic and malic acid at good yields. It can be concluded that the Fe-TS1 catalyst shows a combined effect of the Fe atoms with the specific catalytic effect of the TS-1. Considering TS-1 and Fe-TS1 catalysts, Fe-TS1 gives rise to higher yield results in the first 30 min, being quickly matched by the TS-1. Moreover, the difference in productivity is very slight.

### 3.3 Comparison with Conventional Heating

One of the significant advantages of using MW as a heating source, comparatively with CH is that reactions are usually achieved at milder conditions (e.g., shorter times or lower temperatures) due to the rapid heating process and efficient energy absorption.^29^ Oxidations carried out with the same lignins (IAT and EOL) were compared under MW and CH heating systems, using the same reaction conditions (temperature, pH, lignin concentration, stirring, H_2_O_2_ concentration, and catalyst load).

**Figure 6:**
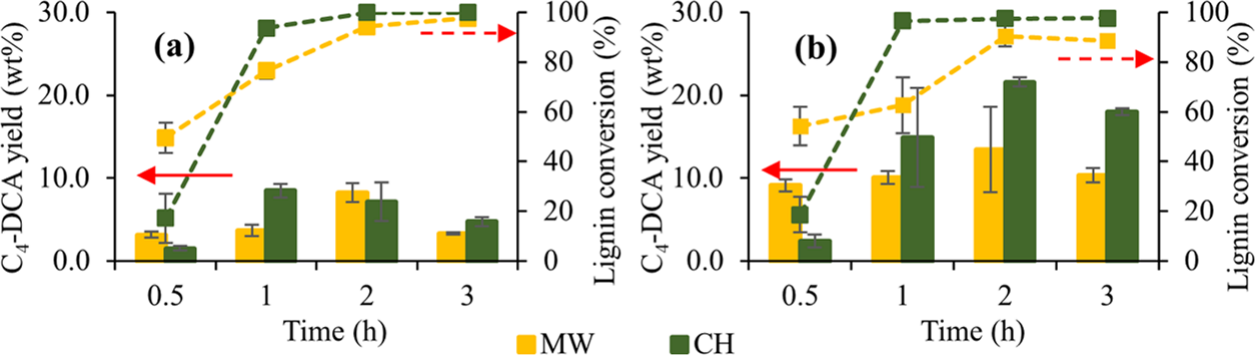
Comparison between microwave (MW) and conventional heating (CH) oxidation for C_4_-DCA yields (columns) and lignin conversion (dashed line) in (a) noncatalyzed and (b) TS-1-catalyzed oxidation (IAT lignin, reaction at 140 °C).

When IAT lignin was oxidized by both methods at 140 °C (10% H_2_O_2_, 10% TS-1 (lignin-basis), 800 rpm stirring), it was found that for both catalyzed and noncatalyzed reactions, the MW heating gave rise to higher C_4_-DCA yields at shorter times. Still, after 1.0 h, the CH becomes more effective, leading to higher yields (Figure 6). The slower heating in the CH process avoided the rapid disproportion of the H_2_O_2_ into H_2_O and O_2_, as observed in the MW reactor during the first minutes. Therefore, the H_2_O_2_ was slowly activated when CH was used, comparatively with the MW, explaining why the conversion was faster in the first 30 min for this later technique. Still, after 1 h, the oxidation was superior for the CH. Moreover, in both CH and MW, it can be seen that C_4_-DCA yields improved with the presence of the TS-1 catalyst.

Since TS-1 is a hydrophobic catalyst with a low amount of OH groups, it does not absorb MW radiation, not being heated directly, but by heat transfer from the solvent, the H_2_O_2,_ and the remaining products in the solution, which is quickly heated by MW.^34^ However, the already adsorbed materials are heated quickly, and desorption from the catalysts occurs faster.^51^ The H_2_O_2_ conversion rate is higher when using MW, so more H_2_O_2_ is decomposed and a lower oxidation efficiency is achieved comparatively with CH.^34^ The H_2_O_2_ activation can go through two mechanisms: the free radical mechanism (releasing of the free ^•^OH radicals in solution) and the interaction with the catalyst to form Ti-OOH species, which interact with the lignin in the catalyst surface. Kooyman et al.’s work showed that the free radical mechanism has a larger contribution in MW than the CH,^34^ which could explain why MW gives rise to a lower lignin conversion, and no considerable increment in the C_4_-DCA yield occurred, even decreasing in some cases. Further studies are needed to confirm this mechanism.

**Figure 7:**
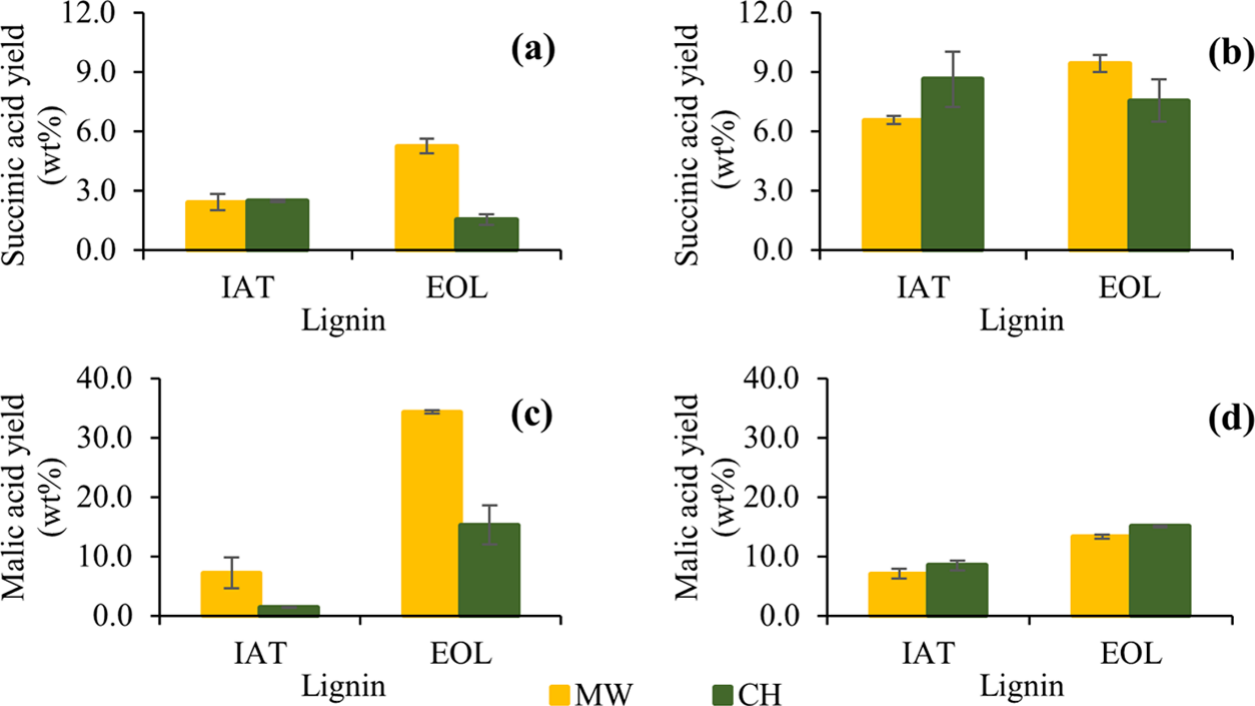
Comparison between microwave (MW) and conventional heating (CH) oxidation using different lignins, for succinic acid (upper level) and malic acid (lower level), in (a, c) noncatalyzed and (b, d) TS-1-catalyzed oxidation (2 h reaction, 160 °C).

Some differences are evident when the two studied lignins are compared using CH and MW at the same conditions (2 h, 160 °C, 800 rpm), as registered in Figure 7. For the noncatalyzed reaction, the succinic acid yield (Figure 7a) was similar for IAT, while for EOL it improved considerably (from 1.6% in CH to 5.2% in MW). Malic acid (Figure 7c) also increased for EOL (from 15.3% in CH to 34.3% in MW) and IAT (from 1.4% in CH to 7.2% in MW). The increase in EOL was evident since the C_4_-DCA yield increased from 16.8% (CH) to 39.6% (MW).

In general, MW enhanced the lignin noncatalyzed oxidation, especially for EOL. It has been reported that MW irradiation enhances the cleavage of β-O-4 bonds^27^ and C_α_–C_β_ bonds,^25^ thus facilitating the conversion of the lignin fractions and monomers to C_4_-DCA. Since EOL has a higher amount of β-O-4 bonds (Table 2), the enhancement is higher for this lignin, explaining why EOL showed a higher C_4_-DCA yield in the noncatalyzed reaction.

In the TS-1 catalyzed reaction, a slight increase in the succinic acid yield for EOL was achieved with the MW reaction (Figure 7b), while malic acid yield was lower for all studied MW conditions (Figure 7d). However, the increasing effect on succinic acid yield caused by TS-1 was maintained in both CH and MW, while the malic acid yield did not suffer any apparent effect in both heating methods.

Regarding the produced compounds and the oxidation mechanism differences, for MW and CH, contradictory evidences considering previously published works were found. In some works, the authors stated that the lignin mechanism of peroxide oxidation does not change when using CH or MW, only the reaction rate is affected.^26^ Other authors suggest that different mechanisms can be found when switching the heating method, namely, by changing the selectivity for specific bonds cleaving.^25,28,29^ However, evidence is scarce since very few works have studied lignin oxidative depolymerization, namely, by comparing MW with CH. Also, the exact conditions in both studies (CH vs MW) were not similar; being possible that the change in the reaction conditions can trigger a modification in the mechanism.^26^

Recently, Qu et al.^26^ performed a study with lignin model compounds using the reaction conditions and by changing the heating system. The authors observed an MW-mediated acceleration in the C_α_–C_β_ bond cleavage of the aromatic compounds, but no acceleration in the aromatic ring cleavage, thus producing the same type of dicarboxylic acids at similar yields. The authors stated that microwave radiation interacts only with intermediates sensitive to electromagnetic waves and that the aromatic ring cleavage was not the case. This conclusion is still based on model compounds. To the best of our knowledge, no experiments were performed with lignins using the same conditions, making it difficult to extrapolate these findings to the complex lignin structure. However, in the present study, it was possible to visualize that EOL oxidation had a strong increase when MW was used (without the catalyst effect), confirming that MW can enhance the production of certain dicarboxylic acids, depending on the lignin structure and the number of labile ether bonds (β-O-4 linkages).

Other published works reported lower C_4_-DCA yields for lignin oxidation by conventional heating, as seen in Table 4, even these experiments were carried out using catalysts to enhance the conversion. The oxidations carried out using TS-1 gave rise to the best succinic acid yields using chalcopyrite (CuFeS_2_),^37^ with the advantage of producing malic acid at a much higher yield with a commercially available catalyst. In this case, the selectivity toward succinic acid was lower, but the overall conversion to C_4_-DCA was higher, especially when using EOL as the feedstock. The effect of MW on lignin peroxide oxidation was also a determinant factor since it enhanced the cleavage of β-O-4 bonds, obtaining excellent malic acid yields in both catalyzed and noncatalyzed oxidations conducted with the organosolv lignins. Summarizing, this work showed that the combination of peroxide oxidation with microwave radiation enhanced the production of C_4_-DCA, and the use of TS-1 helped to increase succinic acid yield.

**Table 4:** C_4_-DCA Yields Reported in Previous Works for Lignin^a^

feedstock	oxidant	catalyst	C_4_-DCA yield (max. value)	ref
alkaline *Populus tremuloides* lignin	O_2_	FeCl_3_	MA 0.06%, FU 12%	(38)
lignin	O_2_	H_3_PW_12_O_4_, H_3_PMo_12_O_4_	SU 1.8%	(52)
lignin (Poplar, Pine, corn stover)	O_2_	LaMn_0.8_Cu_0.2_O_3_	SU < 1%, MAL < 2%	(53)
diluted-acid corn stover, steam-exploded spruce lignin	H_2_O_2_	CuFeS_2_	SU 7%, MAL 0.8%	(36)
lignin	H_2_O_2_	CuFeS_2_	SU 12%, FU 1%, MA 1%	(37)
indulin AT, Lignol, Aldrich alkali lignin, E. globulus kraft lignin	H_2_O_2_	TS-1	SU 11.3%, MAL 19.5%, MA < 1%, FU < 1%	(35)

a SU = succinic acid, FU = fumaric acid, MA = maleic acid, MAL = malic acid.

## 4 Conclusions

The microwave-assisted reaction is an efficient process for lignin peroxide oxidation toward C_4_ dicarboxylic acids. Indulin AT noncatalytic oxidation increased when higher temperatures were used, resulting in a faster obtainment of C_4_-DCA. When TS-1 and Fe-TS1 were used, higher yields of succinic and malic acids were obtained for the catalyzed reactions with EKL and IAT. For EOL, a similar behavior in catalyzed and noncatalyzed reactions was observed for succinic acid but with higher yields for malic acid in the noncatalyzed reaction. Since EOL is an organosolv lignin, the higher amount of β-O-4 bonds allowed a faster oxidation, even in the absence of catalyst, because MW facilitates the cleavage of this linkage. The Fe-TS1 gave rise to faster oxidations than the TS-1 catalyst, given the combined effect of the TS-1 and the Fe atoms deposited on its surface.

When comparing microwave and conventional heating, it was found that microwave enhanced the noncatalytic oxidation of lignins, especially with EOL, whose succinic and malic acid yields were 2 times higher for MW oxidation. However, when TS-1 was used, the MW results were inferior since H_2_O_2_ is easily disproportioned to O_2_, slowing down the oxidative efficiency. This work achieved better results than other previously published works, especially concerning the high yield of malic acid in a noncatalyzed reaction.

Overall, it can be concluded that MW is a very interesting alternative for the future valorization of organosolv lignins. These lignins have bonds easily cleaved when MW is used, leading to a high conversion to added-value products. Also, MW showed good energy efficiency, giving rise to good results for short times. However, it was found that each lignin must be evaluated for both technologies (MW and CH) to confirm the best process to produce the compounds of interest.

## Supporting Information Available

Additional information of GPC calibration curves (Figure S1), lignin calibration curves (Figure S2), DCA calibration curves (Table S1), effect of temperature in formic and acetic acids for noncatalyzed microwave-assisted oxidation of IAT (Figure S3), and evolution of formic and acetic acids through time (Figure S4) (PDF)

## Abbreviations

ATR-FTIR: attenuated total reflectance-Fourier transform infrared spectroscopy
^13^C NMR: ^13^C nuclear magnetic resonance
CH: conventional heating
DCA: dicarboxylic acids
EKL: *E. globulus* kraft lignin
EOL: Lignol organosolv lignin
G: guaiacyl unit
GPC: gel permeation chromatography
H: *p*-hydroxyphenyl unit
IAT: Indulin AT lignin
HPLC: high-performance liquid chromatography
MW: microwave heating
S: syringyl unit
TS-1: titanium silicalite-1

## References

[ref1] Li C.; Zhao X.; Wang A.; Huber G. W.; Zhang T. Catalytic Transformation of Lignin for the Production of Chemicals and Fuels. Chem. Rev. 2015, 115, 11559–11624. 10.1021/acs.chemrev.5b00155. 26479313

[ref2] Kamm B.; Gruber P. R.; Kamm M. Biorefineries-Industrial Processes and Products: Status Quo and Future Directions; Wiley-VCH: Weinheim, 2008; Vol. 1–2.

[ref3] Huang D.; Li R.; Xu P.; Li T.; Deng R.; Chen S.; Zhang Q. The Cornerstone of Realizing Lignin Value-Addition: Exploiting the Native Structure and Properties of Lignin by Extraction Methods. Chem. Eng. J. 2020, 402, 126237 10.1016/j.cej.2020.126237.

[ref4] Berlin A.; Balakshin M. Industrial Lignins. In Bioenergy Research: Advances and Applications; Gupta V. K.; Tuohy M. G.; Kubicek C. P.; Saddler J.; Xu F., Eds.; Elsevier: Amsterdam, 2014; pp 315–336.

[ref5] Rinaldi R.; Jastrzebski R.; Clough M. T.; Ralph J.; Kennema M.; Bruijnincx P. C. A.; Weckhuysen B. M. Paving the Way for Lignin Valorisation: Recent Advances in Bioengineering, Biorefining and Catalysis. Angew. Chem., Int. Ed. 2016, 55, 8164–8215. 10.1002/anie.201510351. PMC6680216 27311348

[ref6] Wang H.; Pu Y.; Ragauskas A.; Yang B. From Lignin to Valuable Products–Strategies, Challenges, and Prospects. Bioresour. Technol. 2019, 271, 449–461. 10.1016/j.biortech.2018.09.072. 30266464

[ref7] Upton B. M.; Kasko A. M. Strategies for the Conversion of Lignin to High-Value Polymeric Materials: Review and Perspective. Chem. Rev. 2016, 116, 2275–2306. 10.1021/acs.chemrev.5b00345. 26654678

[ref8] Cao Y.; Chen S. S.; Zhang S.; Ok Y. S.; Matsagar B. M.; Wu K. C.; Tsang D. C. W. Advances in Lignin Valorization towards Bio-Based Chemicals and Fuels: Lignin Biorefinery. Bioresour. Technol. 2019, 291, 121878 10.1016/j.biortech.2019.121878. 31377047

[ref9] Pandey M. P.; Kim C. S. Lignin Depolymerization and Conversion: A Review of Thermochemical Methods. Chem. Eng. Technol. 2011, 34, 29–41. 10.1002/ceat.201000270.

[ref10] Ma R.; Xu Y.; Zhang X. Catalytic Oxidation of Biorefinery Lignin to Value-Added Chemicals to Support Sustainable Biofuel Production. ChemSusChem 2015, 8, 24–51. 10.1002/cssc.201402503. 25272962

[ref11] Vangeel T.; Schutyser W.; Renders T.; Sels B. F. Perspective on Lignin Oxidation: Advances, Challenges, and Future Directions. Top. Curr. Chem. 2018, 376, 30 10.1007/s41061-018-0207-2. 29974271

[ref12] Rodrigues A. E.; Pinto P. C.; Barreiro M. F.; Esteves da Costa C. A.; Ferreira da Mota M. I.; Fernandes I. An Integrated Approach for Added-Value Products from Lignocellulosic Biorefineries; Springer International Publishing: Cham, 2018; Vol. 288, pp 53–80

[ref13] Costa C. A. E.; Vega-Aguilar C. A.; Rodrigues A. E. Added-Value Chemicals from Lignin Oxidation. Molecules 2021, 26, 4602 10.3390/molecules26154602. 34361756 PMC8346967

[ref14] Werpy T.; Petersen G. Top Value Added Chemicals from Biomass: Volume I—Results of Screening for Potential Candidates from Sugars and Synthesis Gas; National Renewable Energy Lab: Golden, CO, 2004; Vol. 1.

[ref15] Gérardy R.; Debecker D. P.; Estager J.; Luis P.; Monbaliu J.-C. M. Continuous Flow Upgrading of Selected C 2–C 6 Platform Chemicals Derived from Biomass. Chem. Rev. 2020, 120, 7219–7347. 10.1021/acs.chemrev.9b00846. 32667196

[ref16] Behling R.; Valange S.; Chatel G. Heterogeneous Catalytic Oxidation for Lignin Valorization into Valuable Chemicals: What Results? What Limitations? What Trends?. Green Chem. 2016, 18, 1839–1854. 10.1039/c5gc03061g.

[ref17] Palma V.; Barba D.; Cortese M.; Martino M.; Renda S.; Meloni E. Microwaves and Heterogeneous Catalysis: A Review on Selected Catalytic Processes. Catalysts 2020, 10, 246 10.3390/catal10020246.

[ref18] Aguilar-Reynosa A.; Romaní A.; Rodríguez-Jasso R.; Aguilar C. N.; Garrote G.; Ruiz H. A. Microwave Heating Processing as Alternative of Pretreatment in Second-Generation Biorefinery: An Overview. Energy Convers. Manage. 2017, 136, 50–65. 10.1016/j.enconman.2017.01.004.

[ref19] Fan L.; Song H.; Lu Q.; Leng L.; Li K.; Liu Y.; Wang Y.; Chen P.; Ruan R.; Zhou W. Screening Microwave Susceptors for Microwave-Assisted Pyrolysis of Lignin: Comparison of Product Yield and Chemical Profile. J. Anal. Appl. Pyrolysis 2019, 142, 104623 10.1016/j.jaap.2019.05.012.

[ref20] Bartoli M.; Rosi L.; Frediani P.; Frediani M. Bio-Oils from Microwave Assisted Pyrolysis of Kraft Lignin Operating at Reduced Residual Pressure. Fuel 2020, 278, 118175 10.1016/j.fuel.2020.118175.

[ref21] Li H.; Qu Y.; Xu J. Microwave-Assisted Conversion of Lignin. In Production of Biofuels and Chemicals with Microwave; Springer: Dordrecht, 2015; Vol. 3, pp 61–82.

[ref22] Gu X.; He M.; Shi Y.; Li Z. La-Containing Sba-15/H2O2 Systems for the Microwave Assisted Oxidation of a Lignin Model Phenolic Monomer. Maderas: Cienc. Tecnol. 2010, 12, 181–188. 10.4067/S0718-221X2010000300003.

[ref23] Pan J.; Fu J.; Lu X. Microwave-Assisted Oxidative Degradation of Lignin Model Compounds with Metal Salts. Energy Fuels 2015, 29, 4503–4509. 10.1021/acs.energyfuels.5b00735.

[ref24] Zhang D.; Sun B.; Duan L.; Tao Y.; Xu A.; Li X. Photooxidation of Guaiacol to Organic Acids with Hydrogen Peroxide by Microwave Discharge Electrodeless Lamps. Chem. Eng. Technol. 2016, 39, 97–101. 10.1002/ceat.201500251.

[ref25] Zhu G.; Jin D.; Zhao L.; Ouyang X.; Chen C.; Qiu X. Microwave-Assisted Selective Cleavage of CA[Sbnd]CBbond for Lignin Depolymerization. Fuel Process. Technol. 2017, 161, 155–161. 10.1016/j.fuproc.2017.03.020.

[ref26] Qu C.; Ito K.; Katsuyama I.; Mitani T.; Kashimura K.; Watanabe T. Directly Microwave-Accelerated Cleavage of C–C and C–O Bonds of Lignin by Copper Oxide and H2O2. ChemSusChem 2020, 13, 4510–4518. 10.1002/cssc.202000502. 32275119

[ref27] Ouyang X.; Lin Z.; Deng Y.; Yang D.; Qiu X. Oxidative Degradation of Soda Lignin Assisted by Microwave Irradiation. Chin. J. Chem. Eng. 2010, 18, 695–702. 10.1016/S1004-9541(10)60277-7.

[ref28] Ouyang X.; Huang X.; Ruan T.; Qiu X. Microwave-Assisted Oxidative Digestion of Lignin with Hydrogen Peroxide for TOC and Color Removal. Water Sci. Technol. 2015, 71, 390–396. 10.2166/wst.2014.535. 25714638

[ref29] Kim H. G.; Park Y. Manageable Conversion of Lignin to Phenolic Chemicals Using a Microwave Reactor in the Presence of Potassium Hydroxide. Ind. Eng. Chem. Res. 2013, 52, 10059–10062. 10.1021/ie400719v.

[ref30] Li H.; Zhang C.; Pang C.; Li X.; Gao X. The Advances in the Special Microwave Effects of the Heterogeneous Catalytic Reactions. Front. Chem. 2020, 8, 355 10.3389/fchem.2020.00355. 32432084 PMC7216099

[ref31] Panyadee R.; Posoknistakul P.; Jonglertjunya W.; Kim-Lohsoontorn P.; Laosiripojana N.; Matsagar B. M.; Wu K. C. W.; Sakdaronnarong C. Sequential Fractionation of Palm Empty Fruit Bunch and Microwave-Assisted Depolymerization of Lignin for Producing Monophenolic Compounds. ACS Sustainable Chem. Eng. 2018, 6, 16896–16906. 10.1021/acssuschemeng.8b04246.

[ref32] Ouyang X.; Tan Y.; Qiu X. Oxidative Degradation of Lignin for Producing Monophenolic Compounds. J. Fuel Chem. Technol. 2014, 42, 677–682. 10.1016/S1872-5813(14)60030-X.

[ref33] Dai J.; Styles G. N.; Patti A. F.; Saito K. CuSO4/H2O2-Catalyzed Lignin Depolymerization under the Irradiation of Microwaves. ACS Omega 2018, 3, 10433–10441. 10.1021/acsomega.8b01978. 31459170 PMC6645013

[ref34] Kooyman P. J.; Luijkx G. C. A.; Arafat A.; Van Bekkum H. Microwave Heating in the TS-1 Catalyzed Oxyfunctionalisation of n-Hexane. J. Mol. Catal. A: Chem. 1996, 111, 167–174. 10.1016/1381-1169(96)00206-3.

[ref35] Vega-Aguilar C. A.; Barreiro M. F.; Rodrigues A. E. Lignin Conversion into C4 Dicarboxylic Acids by Catalytic Wet Peroxide Oxidation Using Titanium Silicalite-1. Ind. Crops Prod. 2021, 173, 114155 10.1016/j.indcrop.2021.114155.

[ref36] Ma R.; Guo M.; Zhang X. Selective Conversion of Biorefinery Lignin into Dicarboxylic Acids. ChemSusChem 2014, 7, 412–415. 10.1002/cssc.201300964. 24464928

[ref37] Bi Z.; Li Z.; Yan L. Catalytic Oxidation of Lignin to Dicarboxylic Acid over the CuFeS2 Nanoparticle Catalyst. Green Process. Synth. 2018, 7, 306–315. 10.1515/gps-2017-0056.

[ref38] Wu G.; Heitz M. Catalytic Mechanism of Cu2+ and Fe3+ in Alkaline O2 Oxidation of Lignin. J. Wood Chem. Technol. 1995, 15, 189–202. 10.1080/02773819508009507.

[ref39] Lou Y.; Marinkovic S.; Estrine B.; Qiang W.; Enderlin G. Oxidation of Furfural and Furan Derivatives to Maleic Acid in the Presence of a Simple Catalyst System Based on Acetic Acid and TS-1 and Hydrogen Peroxide. ACS Omega 2020, 5, 2561–2568. 10.1021/acsomega.9b02141. 32095680 PMC7033676

[ref40] Vega-Aguilar C. A.; Barreiro M. F.; Rodrigues A. E. Catalytic Wet Peroxide Oxidation of Vanillic Acid as a Lignin Model Compound towards the Renewable Production of Dicarboxylic Acids. Chem. Eng. Res. Des. 2020, 159, 115–124. 10.1016/j.cherd.2020.04.021.

[ref41] Costa C. A. E. E.; Pinto P. C. R.; Rodrigues A. E. Evaluation of Chemical Processing Impact on E. globulus Wood Lignin and Comparison with Bark Lignin. Ind. Crops Prod. 2014, 61, 479–491. 10.1016/j.indcrop.2014.07.045.

[ref42] Costa C.; Alves A.; Pinto P. R.; Sousa R. A.; Borges Da Silva E. A.; Reis R. L.; Rodrigues A. E. Characterization of Ulvan Extracts to Assess the Effect of Different Steps in the Extraction Procedure. Carbohydr. Polym. 2012, 88, 537–546. 10.1016/j.carbpol.2011.12.041.

[ref43] Costa C. A. E.; Pinto P. C. R.; Rodrigues A. E. Lignin Fractionation from E. globulus Kraft Liquor by Ultrafiltration in a Three Stage Membrane Sequence. Sep. Purif. Technol. 2018, 192, 140–151. 10.1016/j.seppur.2017.09.066.

[ref44] Faix O. Fourier Transform Infrared Spectroscopy. In Methods in Lignin Chemistry; Stephen Y. L.; Carlton W. D., Eds.; Springer: Berlin, Heidelberg, 1992; Vol. 8, pp 83–109. 10.1007/978-3-642-74065-7_7.

[ref45] Cateto C. A. B. Lignin-Based Polyurethanes: Characterisation, Synthesis and Applications. Ph.D. Dissertation, University of Porto, Faculty of Engineering: Porto, 2008.

[ref46] Vega-Aguilar C. A.; Barreiro M. F.; Rodrigues A. E. Effect of Methoxy Substituents on Wet Peroxide Oxidation of Lignin and Lignin Model Compounds: Understanding the Pathway to C 4 Dicarboxylic Acids. Ind. Eng. Chem. Res. 2021, 60, 3543–3553. 10.1021/acs.iecr.0c05085.

[ref47] Chan M. N.; Zhang H.; Goldstein A. H.; Wilson K. R. Role of Water and Phase in the Heterogeneous Oxidation of Solid and Aqueous Succinic Acid Aerosol by Hydroxyl Radicals. J. Phys. Chem. C 2014, 118, 28978–28992. 10.1021/jp5012022.

[ref48] Bensalah N.; Louhichi B.; Abdel-Wahab A. Electrochemical Oxidation of Succinic Acid in Aqueous Solutions Using Boron Doped Diamond Anodes. Int. J. Environ. Sci. Technol. 2012, 9, 135–143. 10.1007/s13762-011-0007-5.

[ref49] Dallinger D.; Kappe C. O. Microwave-Assisted Synthesis in Water as Solvent. Chem. Rev. 2007, 107, 2563–2591. 10.1021/cr0509410. 17451275

[ref50] Xia C.; Peng X.; Zhang Y.; Wang B.; Lin M.; Zhu B.; Luo Y.; Shu X. Environmental-Friendly Catalytic Oxidation Processes Based on Hierarchical Titanium Silicate Zeolites at SINOPEC. In Green Chemical Processing and Synthesis; Karamé I., Ed.; InTech, 2017; pp 119–150.

[ref51] Kim K.-J.; Ahn H.-G. The Effect of Pore Structure of Zeolite on the Adsorption of VOCs and Their Desorption Properties by Microwave Heating. Microporous Mesoporous Mater. 2012, 152, 78–83. 10.1016/j.micromeso.2011.11.051.

[ref52] Demesa A. G.; Laari A.; Sillanp M.; Koiranen T. Valorization of Lignin by Partial Wet Oxidation Using Sustainable Heteropoly Acid Catalysts. Molecules 2017, 22, 1625 10.3390/molecules22101625. 28956838 PMC6151388

[ref53] Schutyser W.; Kruger J. S.; Robinson A. M.; Katahira R.; Brandner D. G.; Cleveland N. S.; Mittal A.; Peterson D. J.; Meilan R.; Román-Leshkov Y.; Beckham G. T. Revisiting Alkaline Aerobic Lignin Oxidation. Green Chem. 2018, 20, 3828–3844. 10.1039/C8GC00502H.

