# Supplementary material for: Microwave-Assisted Lignin Wet Peroxide Oxidation to C4 Dicarboxylic Acids
Source: Ind Eng Chem Res. 2022 Mar 4;61(10):3570–81. doi: 10.1021/acs.iecr.1c05004 (PMC9775456; doi:10.1021/acs.iecr.1c05004)
Supplement: Supplementary file 1 — ie1c05004_si_001.pdf [file ie1c05004_si_001.pdf]

Supporting Information for

Microwave-assisted lignin wet peroxide oxidation to C_4 dicarboxylic acids

*Carlos A. Vega-Aguilar[1,2], Carina Costa[1], Maria Filomena Barreiro[2], Alírio E. Rodrigues[1]**

[1] Laboratory of Separation and Reaction Engineering – Laboratory of Catalysis and Materials (LSRE-LCM), Department of Chemical Engineering, Faculdade de Engenharia, Universidade do Porto, Rua Dr. Roberto Frias s/n, 4200-465 Porto, Portugal

[2] Centro de Investigação de Montanha - CIMO, Instituto Politécnico de Bragança, Campus de Santa Apolónia, 5300-253 Bragança, Portugal

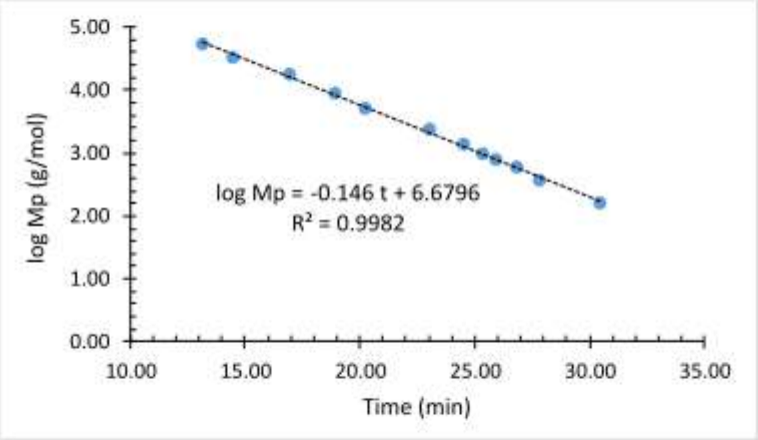

Figure S1. Calibration curves for gel permeation chromatography, using polystyrene standards, by UV spectrophotometric analysis (λ=268 nm). Abbreviations: M_P: peak molecular weight, t: time.

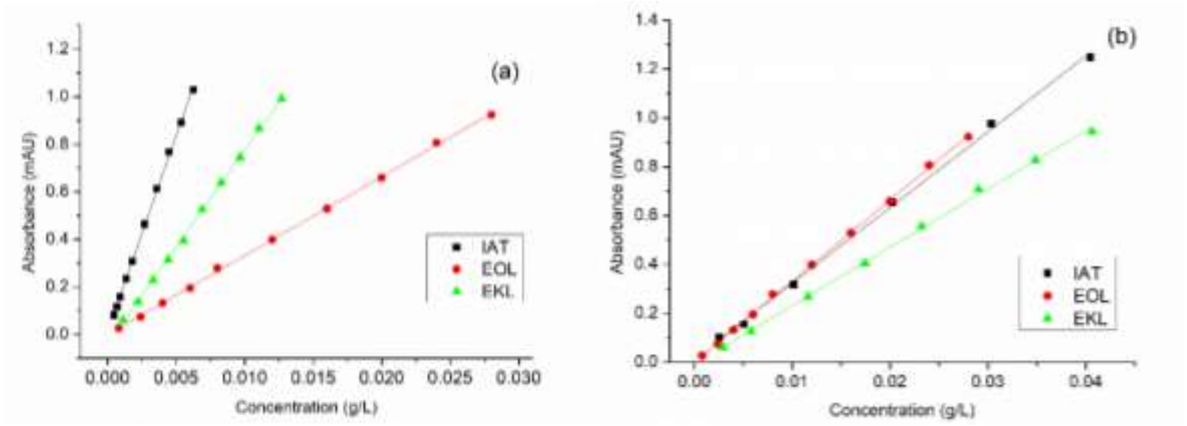

Figure S2. Calibration curves for (a) acid-soluble and (b) insoluble lignin quantification, by UV spectrophotometric analysis (λ=280 nm). Abbreviations: IAT: Indulin AT, EOL: Lignol organosolv lignin, EKL: *E. globulus* kraft lignin.

Table S1. Calibration curves for acid quantification, by HPLC-DAD/RI.

Acid	Time peak (min)	Detection method	C_{start} (mg/L)	C_{final} (mg/L)	Calibration curve			L_D (mg/L)	L_Q (mg/L)
					R^2	*m*	*b*		
Oxalic	9.32	UV 210	10.0	150.0	0.9999	32439	-26896	1.4	4.5
	9.46	RI			0.9997	324.5	-366.5	2.9	10
Maleic	10.72	UV 210	0.247	24.7	0.9999	241540	-13170	0.3	0.8
Tartaric	11.48	UV 210	4.982	400.1	0.9999	3633	-10117	5.7	19
Malic	12.72	UV 210	19.8	990	0.9996	1940	-18054	25	82
	12.87	RI			0.9999	261.3	-714.3	8.8	29
Succinic	15.30	UV 210	4.99	248	0.9977	1321	-8091	14	48
	15.51	RI			0.9997	244.4	-119.0	5.3	18
Formic	17.46	UV 210	50.0	500.0	0.9999	2132	127.1	5.7	19
	17.69	RI			0.9999	111.1	98.95	6.2	21
Fumaric	18.08	UV 210	0.492	24.8	0.9999	335826	54566	0.3	1.0
Acetic	18.80	UV 210	4.945	197.9	0.9951	1473	-3586	16	53
	19.21	RI			0.9996	165.1	89.24	4.5	15

Abbreviations: HPLC-UV/RI: high performance liquid chromatography- diode array detector/refraction index detector; L_D: detection limit, L_Q: quantification limit; *m*: slope; *b*: intercept; C_{start}: inicial concentration; C_{final}: final concentration.

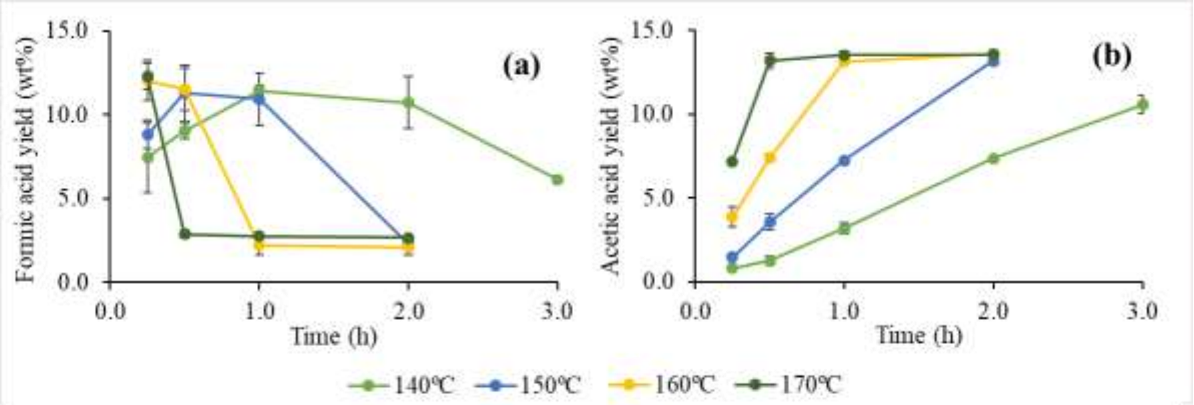

Figure S3 - Non-catalyzed microwave-assisted oxidation of IAT: Effect of temperature on **(a)** formic acid, and **(b)** acetic acid.

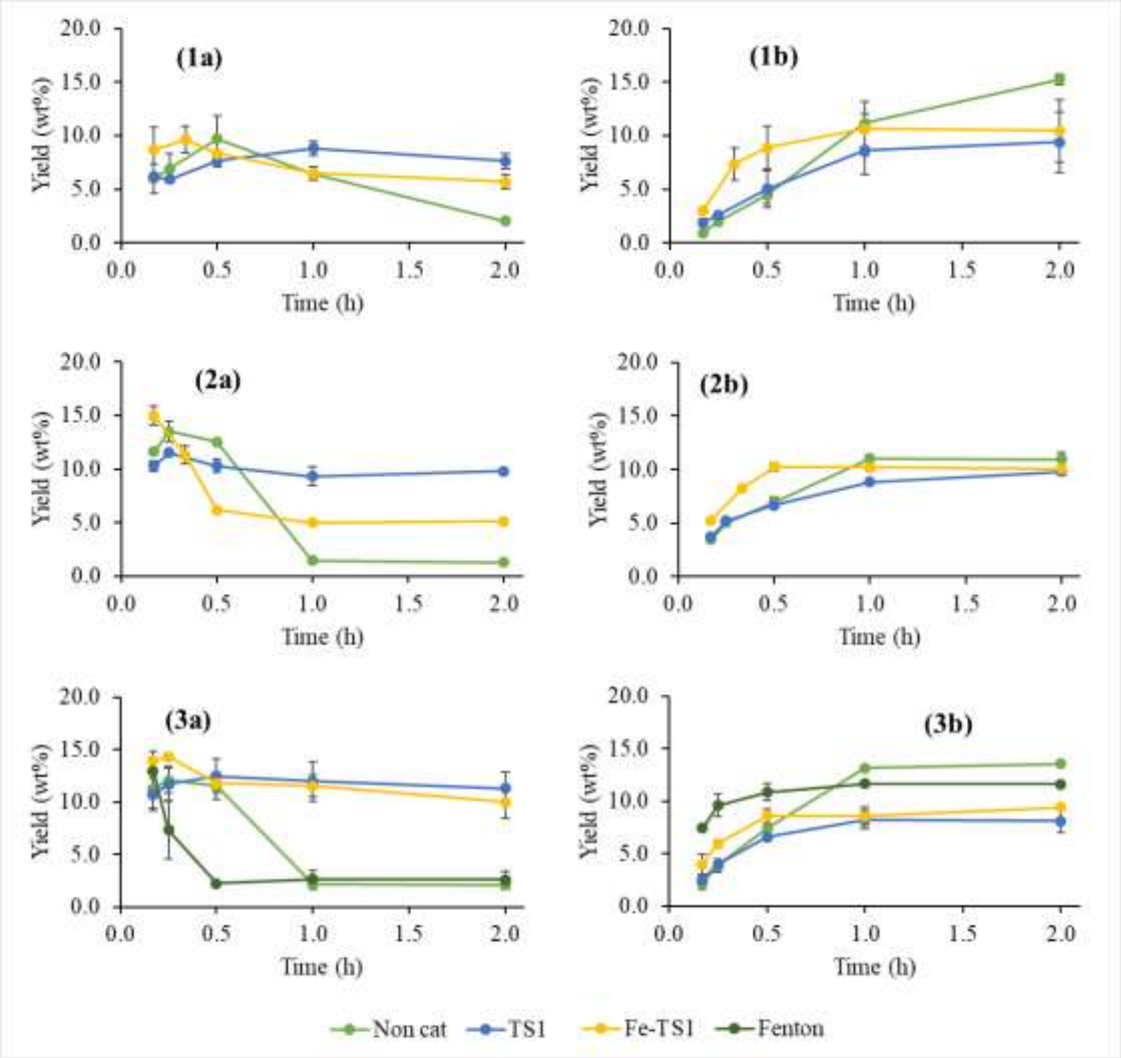

Figure S4 - Evolution of **(a)** Formic acid and **(b)** acetic acid yields through time in the microwave-assisted oxidation for **(1)** EOL, **(2)** EKL, and **(3)** IAT (T=160°C).
